# Acute oral sodium propionate supplementation raises resting energy expenditure and lipid oxidation in fasted humans

**DOI:** 10.1111/dom.13159

**Published:** 2017-12-17

**Authors:** Edward S. Chambers, Claire S. Byrne, Karen Aspey, Yanjie Chen, Saadiyah Khan, Douglas J. Morrison, Gary Frost

**Affiliations:** ^1^ Nutrition and Dietetic Research Group, Section of Investigative Medicine, Faculty of Medicine Hammersmith Campus, Imperial College London UK; ^2^ Stable Isotope Biochemistry Laboratory, Scottish Universities Environmental Research Centre University of Glasgow Glasgow UK

**Keywords:** dietary intervention, energy regulation, randomized trial

## Abstract

Short‐chain fatty acids (SCFAs), produced from fermentation of dietary fibre by the gut microbiota, have been suggested to modulate energy metabolism. Previous work using rodent models has demonstrated that oral supplementation of the SCFA propionate raises resting energy expenditure (REE) by promoting lipid oxidation. The objective of the present study was to investigate the effects of oral sodium propionate on REE and substrate metabolism in humans. Eighteen healthy volunteers (9 women and 9 men; age 25 ± 1 years; body mass index 24.1 ± 1.2 kg/m^2^) completed 2 study visits following an overnight fast. Tablets containing a total of 6845 mg sodium propionate or 4164 mg sodium chloride were provided over the 180‐minute study period in random order. REE and substrate oxidation were assessed by indirect calorimetry. Oral sodium propionate administration increased REE (0.045 ± 0.020 kcal/min; *P* = .036); this was accompanied by elevated rates of whole‐body lipid oxidation (0.012 ± 0.006 g/min; *P* = .048) and was independent of changes in glucose and insulin concentrations. Future studies are warranted to determine whether the acute effects of oral sodium propionate on REE translate into positive improvements in long‐term energy balance in humans.

## 1 INTRODUCTION

A number of studies have shown that increased intake of dietary fibre prevents weight gain and its related metabolic comorbidities.^1^ This association may be attributable to the influence of dietary fibre on the composition and activities of the gut microbiome.^2^ The major end‐products of dietary fibre fermentation by the gut microbiota are the short‐chain fatty acids (SCFAs) acetate, propionate and butyrate,^2^ which have been shown to modulate host metabolism via effects on metabolic pathways and receptor‐mediated mechanisms at different tissue and organ sites.^3^ Specifically, SCFAs act as ligands for G‐protein‐coupled receptors free fatty acid receptor (FFAR)2 and FFAR3, which are expressed throughout the body and modulate energy homeostasis.^4^ The stimulation of FFAR2 on intestinal enteroendocrine L‐cells by SCFAs has been shown to enhance the release and circulating levels of the anorectic hormones peptide YY (PYY) and glucagon‐like peptide‐1 (GLP‐1).^5^ Enhanced colonic SCFA production has therefore been suggested to improve long‐term energy balance by reducing appetite and energy intake.^4^ Our previous work observed that acute oral supplementation with an inulin‐propionate ester, designed to specifically increase delivery of propionate to the human colon, reduced energy intake, which was associated with enhanced PYY and GLP‐1 release.^6^ In a follow‐up investigation, daily oral supplementation with inulin‐propionate ester for 24 weeks prevented body weight gain and visceral adipose tissue accumulation in overweight adults^6^; however, there was no detectable change in anorectic gut hormone release or energy intake after long‐term colonic propionate delivery, suggesting that gut‐derived propionate has positive effects on energy balance and body weight, independently of alterations in energy intake. Indeed, a number of studies have shown that mice receiving a gut microbial transplant that promotes caecal propionate production display no changes in energy intake, yet body composition and glycaemic control are improved.^7^ Furthermore, oral administration of sodium propionate in mice has also been shown to prevent gains in body weight without reducing energy intake.^8^, ^9^ Orally supplemented SCFAs would be rapidly absorbed from the upper gastrointestinal tract, suggesting that propionate could affect energy metabolism by entering the portal and systemic circulation. An intraperitoneal injection of propionate in mice was found to promote sympathetic nervous system activity, via stimulation of FFAR3 expressed at the level of the sympathetic ganglion, elevating heart rate and energy expenditure.^10^ The addition of sodium propionate to the feed of mice has also been reported to protect against diet‐induced obesity by enhancing energy expenditure via a promotion of lipid oxidation in hepatic and adipose tissue.^8^

There is currently limited in vivo evidence to support an effect of gut‐derived propionate on energy expenditure and substrate oxidation in humans. Nevertheless, a recent report in overweight and obese men showed that a rectal infusion of a SCFA mixture high in propionate increased fasting resting energy expenditure (REE) by stimulating lipid oxidation.^11^ In the present study we tested the hypothesis that acute oral intake of sodium propionate would increase REE and rates of lipid oxidation.

## 2 METHODS

All subjects provided informed, written consent to participate in the study, which was approved by the Wales Research Ethics Committee 6 (15/WA/0415) and carried out in accordance with the Declaration of Helsinki. Men and women aged 18 to 65 years, with a body mass index of 18 to 35 kg/m^2^ were recruited. A detailed methodology and protocol schematic is presented in the File S1 and Figure S1 in File S1.

Volunteers completed 2 study visits separated by >2 days. The mean ± SD (range) period between study visits was 5 ± 2 (2‐10) days. Volunteers were requested to refrain from strenuous exercise and alcohol consumption and to consume a standard evening meal prior to fasting overnight for >10 hours before the study visits.

Volunteers arrived at the National Institute of Health Research (NIHR) Imperial Clinical Research Facility between 8:00 and 9:00 am and were asked to void their bladder, and body weight was then recorded to the nearest 0.1 kg. Urine was collected for the remainder of the study visit to provide an estimate of urinary nitrogen excretion from urea concentration.^12^ A cannula was inserted into an antecubital vein and 2 fasting blood samples were collected >5 minutes apart. Further blood samples were taken at 60, 120 and 180 minutes.

In a randomized, double‐blind, crossover design, volunteers were provided with tablets containing 1369 mg sodium propionate (propionate trial) or 833 mg sodium chloride (sodium‐matched control trial) at 0, 30, 60, 90 and 120 minutes. The total amount of sodium propionate ingested over the study visit was 6845 mg (71 mmol). The propionate and control tablets (Table S1 in File S1) had an enteric coating (Acryl‐EZE; Colorcon, Dartford, UK), resistant to the pH of gastric fluid, that readily dissolves at a pH >5.5.^13^

Volunteers lay semi‐recumbent under an indirect calorimeter canopy (GEM Nutrition, Daresbury, UK) between −20 and 0 minutes, 40 and 60 minutes, 100 and 120 minutes and 160 and 180 minutes. The calorimeter was calibrated with “zero” (0.00% O_2_ and 0.00% CO_2_) and “span” (20.00% O_2_, 1.00% CO_2_) gases (BOC Gases, Guildford, UK) and REE, respiratory exchange ratio (RER) and carbohydrate and lipid oxidation rates were calculated at each time point from mean values of VO_2_ and VCO_2_, with adjustment for urinary nitrogen excretion.^12^, ^14^ Heart rate and blood pressure were measured at 0, 60, 120 and 180 minutes (SureSigns VM4, Phillips, Cambridge, Massachusetts). Volunteers were asked to complete 100‐mm visual analogue scales to assess subjective hunger and nausea at 0, 30, 60, 90, 120, 150 and 180 minutes.

## 3 RESULTS

**Figure 1:**
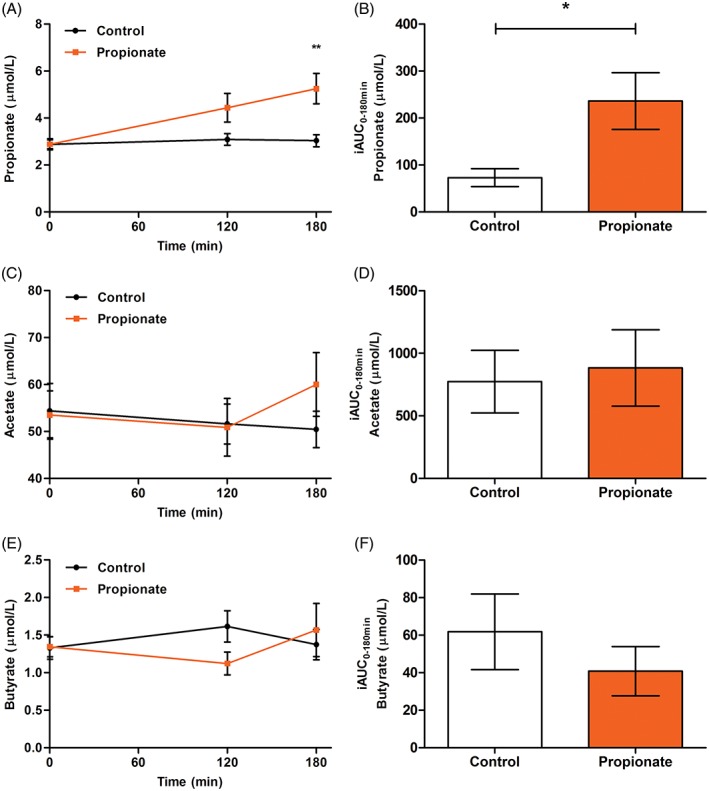
The effect of oral sodium propionate supplementation on short chain fatty acid levels in peripheral blood. A, Propionate (time × trial *P* = .043) and B, Propionate incremental area under the curve (iAUC; *P* = .021). C, Acetate (time × trial *P* = .209) and D, Acetate iAUC (*P* = .761). E, Butyrate (time × trial *P* = .272) and F, Butyrate iAUC (*P* = .587). All data expressed as mean ± SEM (*n* = 18)

Data were analysed from 18 volunteers who completed the 2 study visits (9 women and 9 men; age 25 ± 1 years; body mass index 24.1 ± 1.2 kg/m^2^) and no significant differences were found between trials in baseline values for all measurements (Table S2 in File S1). Levels of propionate in peripheral blood were higher in the propionate trial (Figure 1A,B; mean difference 1.19 ± 0.34 μmol/L; effect of trial *P* = .003), with values significantly raised at 180 minutes compared with control (3.04 ± 0.26 μmol/L vs 5.25 ± 0.65 μmol/L; *P* = .005). There were no differences in circulating levels of acetate (Figure 1C,D; mean difference 2.64 ± 3.99 μmol/L; effect of trial *P* = .518) or butyrate (Figure 1E,F; mean difference −0.09 ± 0.21 μmol/L; effect of trial *P* = .657) between trials.

**Figure 2:**
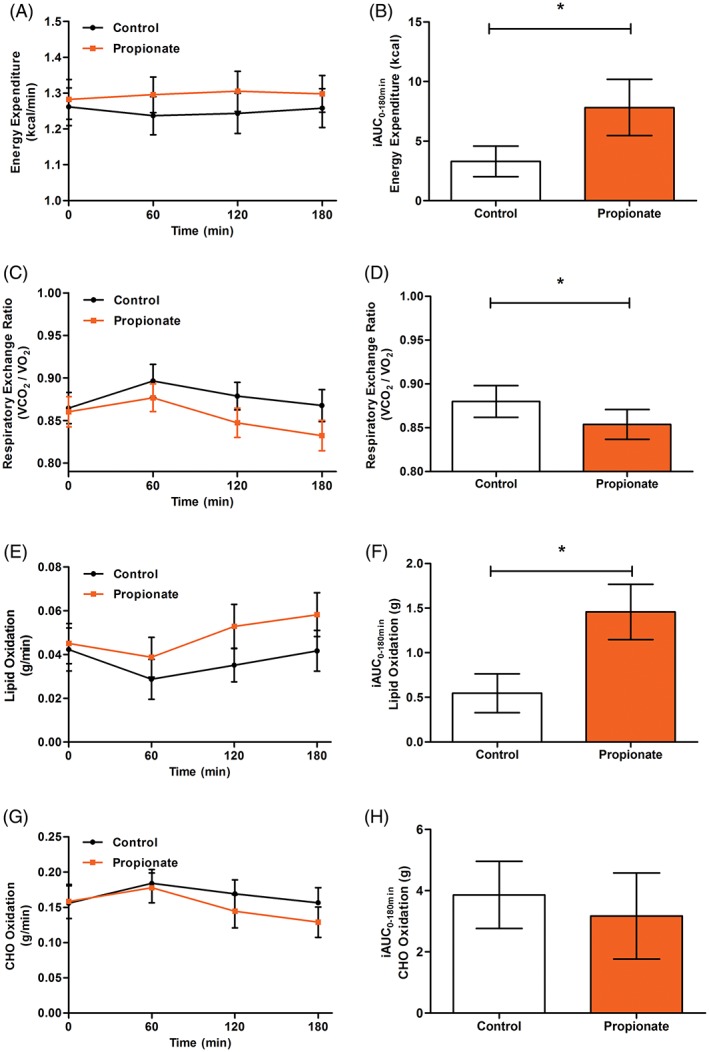
Effect of oral sodium propionate supplementation on resting energy expenditure and substrate oxidation. A, Resting energy expenditure (REE; time × trial *P* = .388) and B, REE incremental area under the curve (iAUC; *P* = .036). C, Respiratory exchange ratio (RER; time × trial *P* = .158) and D, Mean RER (*P* = .040). E, Lipid oxidation (time × trial *P* = .075) and F, Lipid oxidation iAUC (*P* = .019). G, Carbohydrate oxidation (time × trial *P* = .199) and H, Carbohydrate oxidation iAUC (*P* = .338). All data expressed as mean ± SEM (*n* = 18)

The REE was significantly higher after propionate compared with control (Figure 2A,B), with a mean difference of 0.045 ± 0.020 kcal/min (effect of trial *P* = .036). The increase in REE was accompanied by a significantly lower RER in the propionate trial (Figure 2C,D; 0.88 ± 0.02 vs 0.85 ± 0.02; effect of trial *P* = .040), with higher lipid oxidation rates compared with control supplementation (Figure 2E,F), with a mean difference of 0.012 ± 0.006 g/min (effect of trial *P* = .048). There were no differences in rates of carbohydrate oxidation (Figure 2G,H; mean difference −0.014 ± 0.13 g/min; effect of trial *P* = .283) or protein oxidation (control 0.062 ± 0.006 g/min vs propionate 0.059 ± 0.005 g/min; *P* = .535) between trials.

The effects of propionate on energy homeostasis were not related to differences in circulating levels of glucose (Figure S2A,B in File S1; mean difference 0.4 ± 0.9 mmol/L; effect of trial *P* = .666) or insulin (Figure S2C,D in File S1; mean difference −0.1 ± 0.9 μU/mL; effect of trial *P* = .742). There was no mean difference between trials in levels of non‐esterified fatty acids (NEFA; Figure 2E,F; mean difference −0.02 ± 0.05 mmol/L; effect of trial *P* = .767), but there was a significant time × trial interaction (*P* = .017). Post hoc tests revealed no differences at any time point, with a trend for NEFA levels to be lower in the propionate trial at 180 minutes (0.686 ± 0.057 mmol/L vs 0.595 ± 0.049 mmol/L; *P* = .082). There were no significant differences in glycerol concentration (Figure S4A in File S1; mean difference − 0.5 ± 3.0 μmol/L; effect of trial *P* = .865) between study visits. PYY levels were measured at baseline and 180 minutes, and a significant time × trial interaction (*P* = .039) revealed raised concentration after propionate at 180 minutes compared with control (33.6 ± 7.7 pmol/L vs 56.9 ± 8.6 pmol/L; *P* = .012).

There were no significant differences in heart rate (Figure S5A in File S1; mean difference 1.0 ± 0.8 bpm; effect of trial *P* = .197) or mean arterial blood pressure (Figure S5B in File S1; mean difference 1.0 ± 0.9 mm Hg; effect of trial *P* = .267) between study visits. Volunteers reported no differences in subjective hunger between trials (Figure S6A in File S1; mean difference −2.2 ± 2.4 mm; effect of trial *P* = .370), yet subjective nausea was significantly raised in the propionate trial (Figure S6B in File S1; mean difference 3.2 ± 1.2 mm; effect of trial *P* = .013).

## 4 DISCUSSION

Previous work conducted in mice has reported increased energy expenditure with the addition of sodium propionate to the diet.^8^ A direct translation of these findings from murine models to humans should not be assumed, primarily because of substantial species differences in whole‐body REE (expressed per unit mass) and the relative size and contribution of different tissue and organ sites to REE;^15^ however, in the present study we did observe that acute oral administration of 6845 mg sodium propionate increased REE in overnight fasted humans independently of changes in glucose or insulin levels. The present study also corroborates a recent report in overweight humans that rectal infusion of an SCFA mixture high in levels of propionate enhances REE in the fasted state.^11^ A common finding in these murine and human investigations is that propionate increased REE by promoting the rates of whole‐body lipid oxidation. In the present study, we observed an increase in the peripheral concentrations of propionate in peripheral blood after oral sodium propionate supplementation. Previous investigations in humans would indicate that the majority (>90%) of propionate absorbed from the gastrointestinal tract is extracted and metabolized by the liver before entering the systemic circulation.^16^ This suggests that the changes in REE and substrate handling recorded in the present investigation could be primarily at the hepatic level. Indeed, work using hepatic tissue from SCFA‐fed mice has demonstrated that propionate increases the expression of mitochondrial uncoupling protein 2 (UCP2) and the ratio of AMP:ATP, thus inhibiting lipogenesis and stimulating rates of β‐oxidation.^8^ This may provide a metabolic explanation for our previous observation that long‐term colonic propionate delivery reduced intrahepatocellular lipid content in adults with non‐alcoholic fatty liver disease.^6^ Nevertheless, the increased levels of propionate in peripheral blood could also modulate metabolic processes to promote lipid oxidation at other organ sites, including adipose tissue and skeletal muscle.^3^ Further work is required to fully elucidate the contribution of different organ sites to the observed effects of oral sodium propionate on whole‐body REE and lipid oxidation.

Unlike a previous investigation performed in mice,^10^ we did not record an effect of propionate on sympathetic nervous system activity, as measured by changes in heart rate and blood pressure. This may be explained by the much larger dose and route of administration of propionate (1 g/kg body weight by intraperitoneal injection) in the rodent study. In humans, daily propionate production is estimated at 29.5 mg/kg body weight,^4^ thus the acute dose of sodium propionate provided in the present study represents a substantial increase in habitual propionate absorption from the gut (2089 ± 106 mg/d calculated from volunteer body weights). The present methodology is unable to differentiate the contribution of exogenous and endogenous sources to the recorded increase in whole‐body lipid oxidation, however, indirect evidence would indicate this is unlikely to be explained solely by oxidation of the ingested propionate. Propionate oxidation produces an RER of 0.86, which is higher than that recorded throughout the propionate trial, suggesting an enhanced oxidation of endogenous lipids that yield a lower RER. We also observed that the intervention affected endogenous lipid metabolism, as shown by the significant effect on NEFA levels, with post hoc tests demonstrating a trend for a reduction in circulating NEFA at 180 minutes when systemic propionate levels were raised. The stimulation of FFAR2 expressed on adipose tissue has previously been shown to inhibit lipolysis and the levels of circulating NEFA^17^ and to stimulate lipid expenditure in other tissues, including muscle and liver.^18^ A recent stable isotope study in humans has also shown that a portion of gut‐derived propionate is utilised in hepatic gluconeogenesis rather than being directly oxidized.^19^ The fraction of propionate incorporated into glucose was reported to be relatively small (6%), but the postprandial study conditions employed may underestimate the amounts of propionate used for gluconeogenesis in the overnight fasted conditions of the present study. Rates of gluconeogenesis increase throughout the post‐absorptive state^20^ and it is feasible that, after >12 hours of fasting, greater amounts of propionate would be used to sustain the higher rates of endogenous glucose production.

Propionate levels became significantly increased in peripheral blood at 180 minutes; thus, it is unlikely that the complete absorption and bioavailability of the ingested sodium propionate was measured in the present study protocol. It would be of interest to determine how long the metabolic effects of the oral sodium propionate dose persist in the fasted state and the impact on energy metabolism in other physiological states, including postprandially and during physical activity. The maximum ATP yield from propionate oxidation is 18 ATP/mol, equivalent to 131.4 kcal/mol.^21^ Assuming all propionate was oxidized, the ingested sodium propionate had a maximum energy recovery of 9.3 kcal, and thus the observed 0.045 kcal/min increase in REE would need to be maintained for >3.5 hours to achieve a negative energy balance. The intervention may also have influenced energy balance by reducing energy intake, as we have previously observed that acutely increasing colonic propionate levels reduces ad libitum food intake.^6^ Indeed, we observed an increase in the anorectic gut hormone PYY at the end of the study period that may have modified energy intake had it been quantified. Consequently both energy expenditure and intake would need to be measured to determine the extent to which oral sodium propionate administration acutely modulates energy balance. This would provide evidence of the utility of sodium propionate as an intervention to improve chronic energy balance and body weight management in humans.

In summary, the present study provides the first direct evidence that acute oral administration of sodium propionate stimulates REE and whole‐body lipid oxidation in humans, thus corroborating the outcome of investigations conducted in mice.^8^ Murine models show that chronic sodium propionate ingestion promotes benefits to body weight and glucose homeostasis^8^, ^9^ and long‐term studies are needed to demonstrate the same efficacy in humans.

## Supporting information


**File S1.** Supplementary Material.
