# Supplementary material for: Acute oral sodium propionate supplementation raises resting energy expenditure and lipid oxidation in fasted humans
Source: Diabetes Obes Metab. 2017 Dec 17;20(4):1034–9. doi: 10.1111/dom.13159 (PMC5873405; doi:10.1111/dom.13159)
Supplement: Supplementary file 1 — File S1. Supplementary Material. [file DOM-20-1034-s001.docx]

***SUPPLEMENTARY MATERIAL***

***METHODS***

Potential participants were recruited from the Hammersmith and South Kensington campuses of Imperial College London through poster advertisements. Individuals were excluded if they met any of the following criteria: clinically significant illness (including type 1 or type 2 diabetes), medication known to affect energy homeostasis, antibiotic medication, a weight loss of 3 kg or greater in the preceding two months, smoking, substance abuse, psychiatric illness, and any abnormalities detected on physical examination, electrocardiography, or screening blood tests (measurement of complete blood count and fasting glucose). Women were ineligible if they were pregnant or breast-feeding. A urinary pregnancy test was conducted at screening and before the collection of experimental data at the two study visits.

To avoid the possible effect of the menstrual cycle on energy metabolism [^1^](#_ENREF_1), female participants were asked to complete the two study visits <7 days apart during the early phase of the menstrual cycle (days 1–7). Participants were asked to eat the same ready-made or shop-prepared meal the evening before each study visit. Participants were free to choose their meal and compliance was assessed by requesting that volunteers bring the outer packaging of the meal and the receipt of purchase to the study visit.

During the measurements of resting energy expenditure (REE) and substrate oxidation, participants rested on beds and relaxed, but were not permitted to sleep. In the intervals between measurements, physical activity was restricted and subjects remained rested on the beds, but were allowed to read and listen to personal music devices.

The sodium propionate and sodium chloride tablets were prepared by Quay Pharma (UK). The components of the tablets are presented in Table S1. The enteric coating (Acryl-EZE, Colorcon, UK)[^2^](#_ENREF_2) added ~11% to the overall tablet weight. The tablets complied with the Ph Eur disintegration test for gastro-resistant tablets. Less than 10% of the tablet disintegrates after 120 min in 0.1M HCl. With the replacement of the 0.1M HCl with pH 6.8 phosphate buffer solution the tablet then disintegrates within 60 min.

Plasma glucose was measured from blood collected into sodium fluoride plasma tubes and assayed using the Infinity Hexokinase Reagent (Thermo Scientific, USA) measured on a Spectramax I3X plate reader (Molecular Devices, USA). Insulin-like immunoreactivity was measured from blood collected into serum-separating tubes and quantified using an ultra-sensitive human insulin radioimmunoassay (RIA) (Millipore, USA). Serum short chain fatty acids (SCFAs) were measured with the use of an Agilent 7000C Triple Quadrupole GC/MS System according to a previously published method [^3^](#_ENREF_3). Serum non-esterified fatty acids (NEFA) and glycerol were measured using colorimetric assays (Randox, UK) using an ELx808™ Absorbance Microplate Reader (BioTek, USA). 2 mL blood was collected into heparin-coated tubes containing 40µL of Aprotinin (Bayer, UK) and peptide YY (PYY) measured using an established inhouse RIA [^4^](#_ENREF_4)

Participants completed two 100 mm visual analogue scales (VAS) at each time point asking *“How hungry do you feel right now?”* and *“How sick do you feel right now?”* Each VAS was anchored with *“Not at all”* and *“Extremely”* at 0mm and 100mm, respectively.

***Calculations and statistical analysis***

A power calculation confirmed that 16 participants would be sufficient to detect a 7.5% difference in the primary outcome, REE, with a standard deviation (SD) of 10 %(α=0.05, power=0.80). 21 participants were recruited to allow a dropout rate of 25%. Participants’ were excluded from data analysis if the coefficient of variation (CV) of their baseline REE from the two study visits was >10%. Under standard conditions (measured following a >8h fast and abstinence from strenuous exercise), the reported within-subject CV in REE is 3-8%[^5^](#_ENREF_5). A CV >10% could indicate measurement error or a failure of volunteers to comply with the requested standard evening meal, overnight fast and refrainment from strenuous physical activity [^6^](#_ENREF_6). A summary of recruitment and retention is presented in Figure S7. The mean ± SD (range) within-subject CV of baseline REE for the 18 volunteers assessed was 3.3±2.5 (0.4-7.8) %.

Time course data was analysed by repeated measures ANOVA, with time and treatment as within-subject variables. Post hoc Fishers LSD tests were used when a significant interaction was identified. The positive incremental area under the curve (iAUC) was calculated as the area above baseline with the use of the trapezoid rule. iAUC values were calculated using GraphPad Prism Version 5.0. Data were checked for normality using the Shapiro-Wilk Test. All iAUC data was not normally distributed and therefore compared with nonparametric Wilcoxon signed rank tests. All statistical analyses were conducted with SPSS version 23.0 for Windows (SPSS Inc, USA). Data are presented as means ± SEM and P<0.05 was considered significant.

***TABLES***

**Table S1. The components of a sodium propionate and sodium chloride tablet.**

|  |  |  |
| --- | --- | --- |
|  | **Amount (mg)** | |
| **Component** | **Control** | **Propionate** |
| **Sodium Propionate** |  | 684.5 |
| **Sodium Chloride** | 416.4 |  |
| **Kollidon K90** |  | 6.9 |
| **Avicel pH 102** | 523.6 | 248.6 |
| **Magnesium Stearate** | 10 | 10 |
| **Kollidon VA64** | 50 | 50 |
| **Total** | 1000 | 1000 |
|  |  |  |

**Table S2. Baseline values of measurements in the Control and Propionate trials.**

|  |  |  |  |
| --- | --- | --- | --- |
|  | **Control** | **Propionate** | **P Value** |
| **Body Weight (kg)** | 70.8±3.6 | 70.8±3.6 | 0.874 |
| **Resting Energy Expenditure (kcal/min)** | 1.262±0.052 | 1.282±0.056 | 0.269 |
| **Respiratory Exchange Ratio**  **(VCO_2_/VO_2_)** | 0.865±.018 | 0.860±.018 | 0.778 |
| **Lipid Oxidation (g/min)** | 0.042±0.010 | 0.045±0.009 | 0.689 |
| **Carbohydrate Oxidation (g/min)** | 0.158±0.025 | 0.156±0.024 | 0.888 |
| **Propionate (µmol/L)** | 2.88±0.23 | 2.88±0.19 | 0.955 |
| **Acetate (µmol/L)** | 54.43±5.80 | 53.51±5.14 | 0.841 |
| **Butyrate (µmolL)** | 1.33±0.15 | 1.35±0.13 | 0.940 |
| **Glucose (mmol/L)** | 4.7±0.1 | 5.0±0.1 | 0.065 |
| **Insulin (µU/mL)** | 10.1±0.7 | 10.1±0.6 | 0.862 |
| **NEFA**  **(mmol/L)** | 0.548±0.057 | 0.532±0.053 | 0.793 |
| **Glycerol**  **(µmol/L)** | 60.5±5.0 | 60.2±4.8 | 0.952 |
| **PYY**  **(pmol/L)** | 42.8±8.2 | 37.6±5.8 | 0.563 |
| **Heart Rate (bpm)** | 62.5±1.6 | 62.8±1.5 | 0.728 |
| **Mean Arterial Pressure (mmHg)** | 80.4±2.2 | 80.4±2.0 | 0.974 |
| **Hunger VAS (mm)** | 47±4 | 42±4 | 0.060 |
| **Nausea VAS (mm)** | 6±3 | 7±3 | 0.415 |
|  |  |  |  |

All data expressed as mean ± SEM.P values calculated from paired t-tests.

***FIGURES***

***
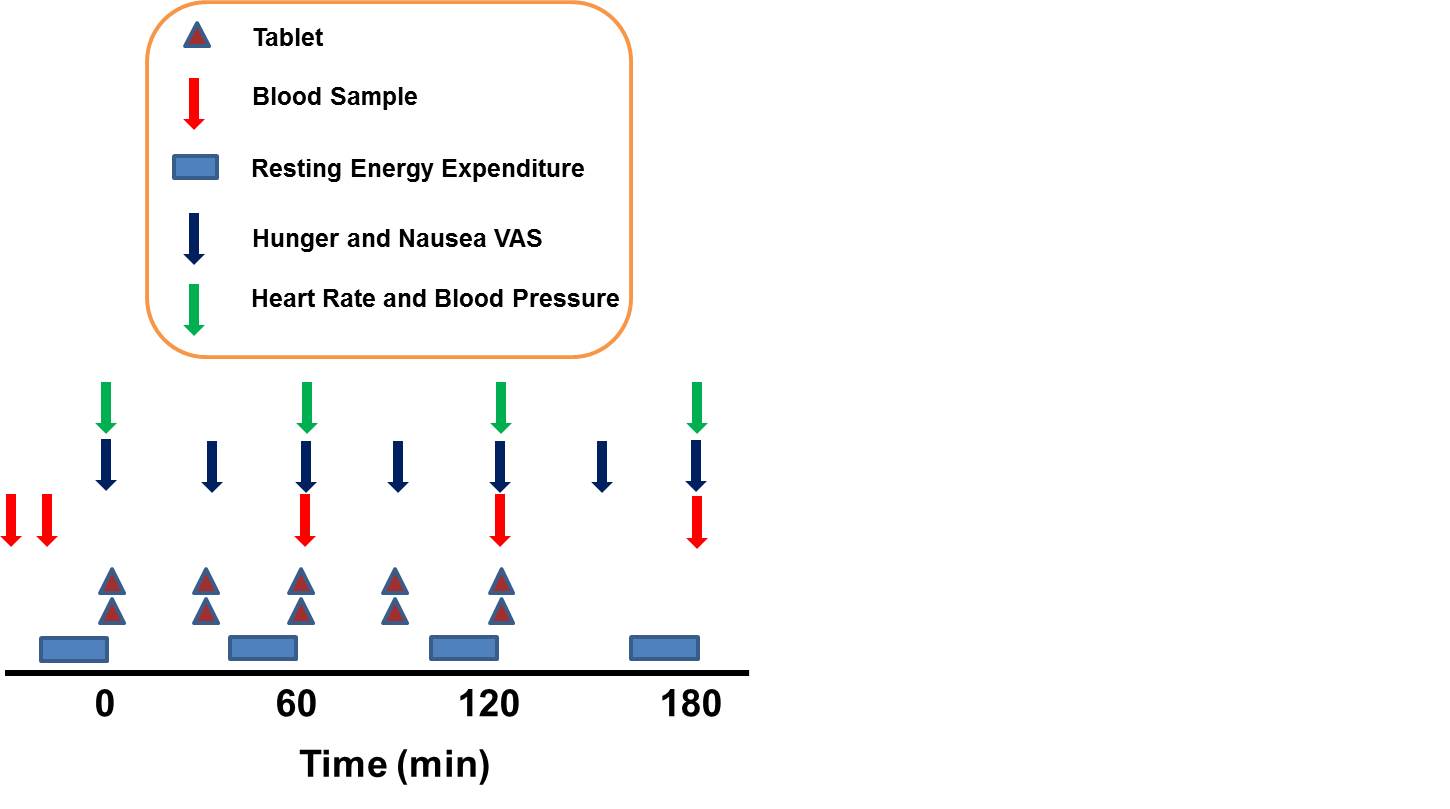
***

**Figure S1. The study protocol.**

**
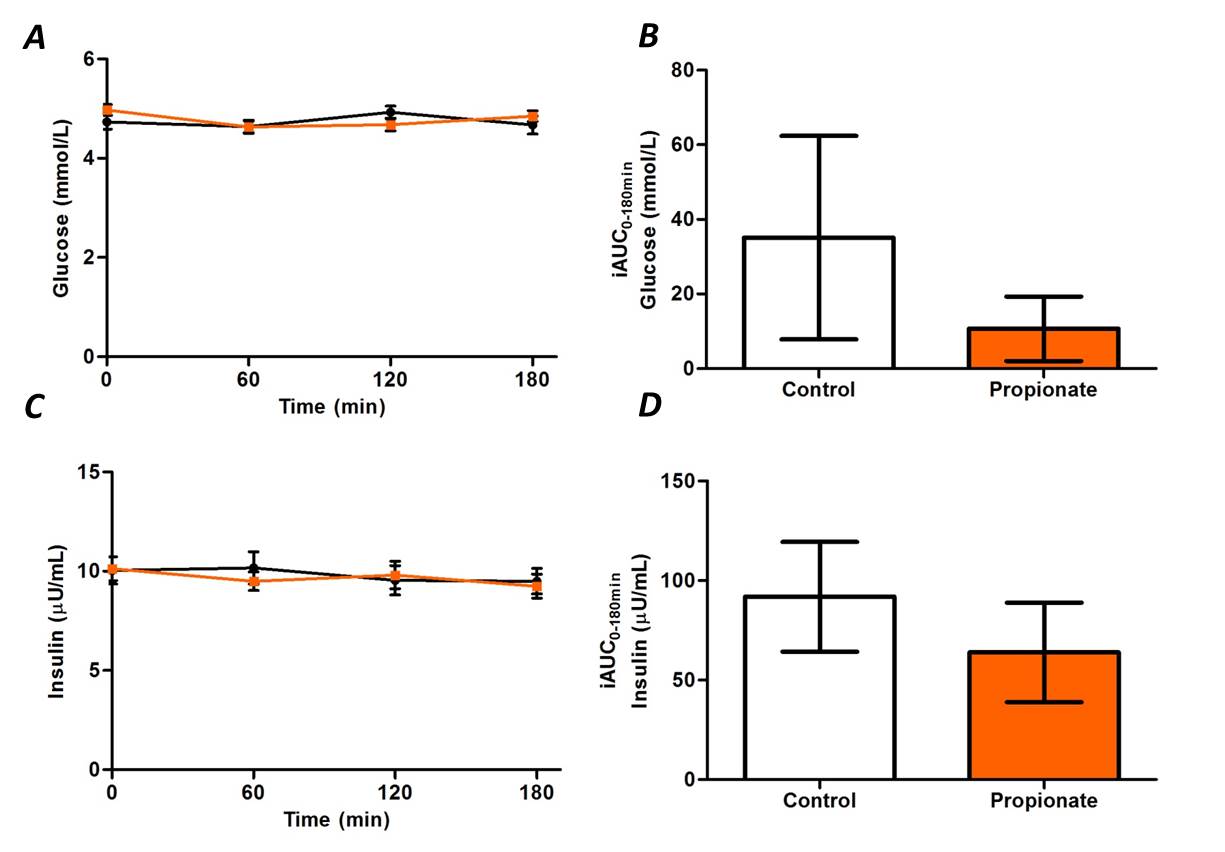
**

**Figure S2. The effect of oral sodium propionate supplementation on glucose and insulin levels in peripheral blood. *A.*** Glucose (Time×Trial: P= 0.112) and ***B.*** Glucose iAUC (P=0.135). ***C.*** Insulin (Time×Trial: P= 0.494) and ***D.*** Insulin iAUC (P=0.293). All data expressed as mean ± SEM (n=18).

**
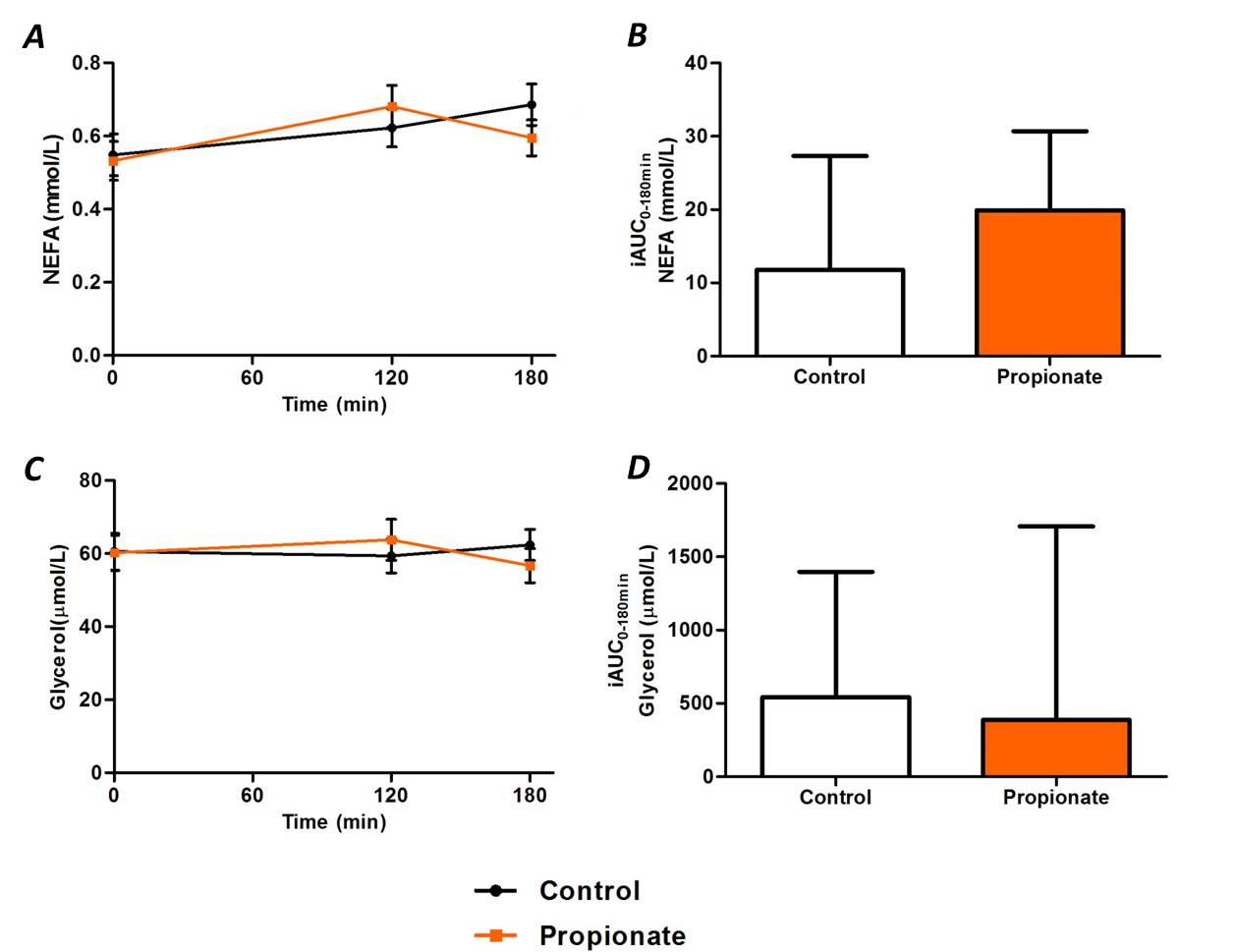
**

**Figure S3. The effect of oral sodium propionate supplementation on non-esterified fatty acids (NEFA) and glycerol levels in peripheral blood. *A.*** NEFA (Time×Trial: P= 0.017) and ***B.*** NEFA iAUC (P=0.288). ***C.*** Glycerol (Time×Trial: P= 0.303) and ***D.*** Glycerol iAUC (P=0.897). All data expressed as mean ± SEM (n=18).


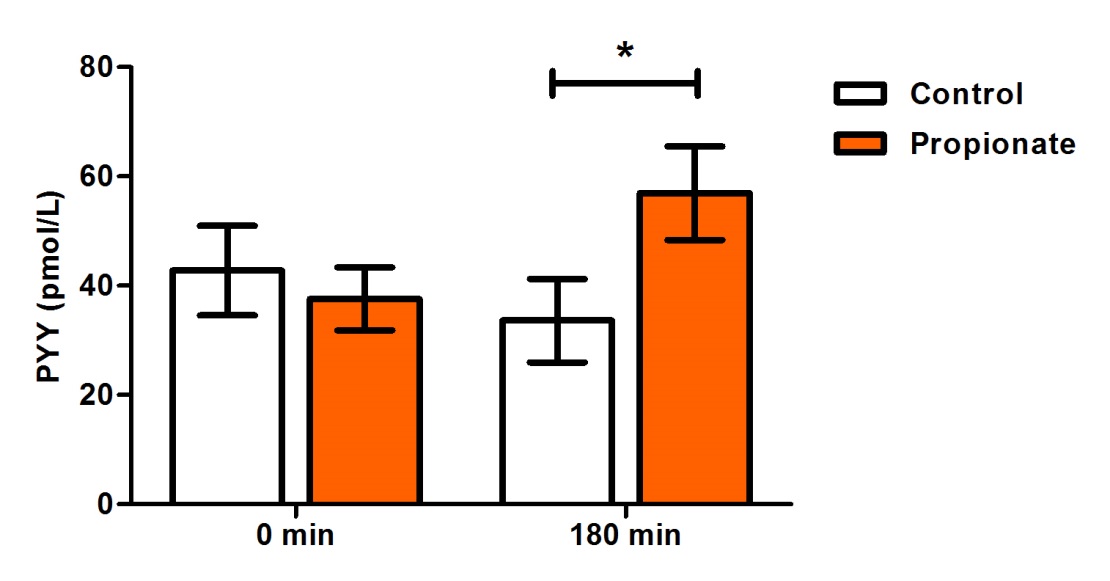


**Figure S4. The effect of oral sodium propionate supplementation on PYY levels in peripheral blood.** All data expressed as mean ± SEM (n=18).

**
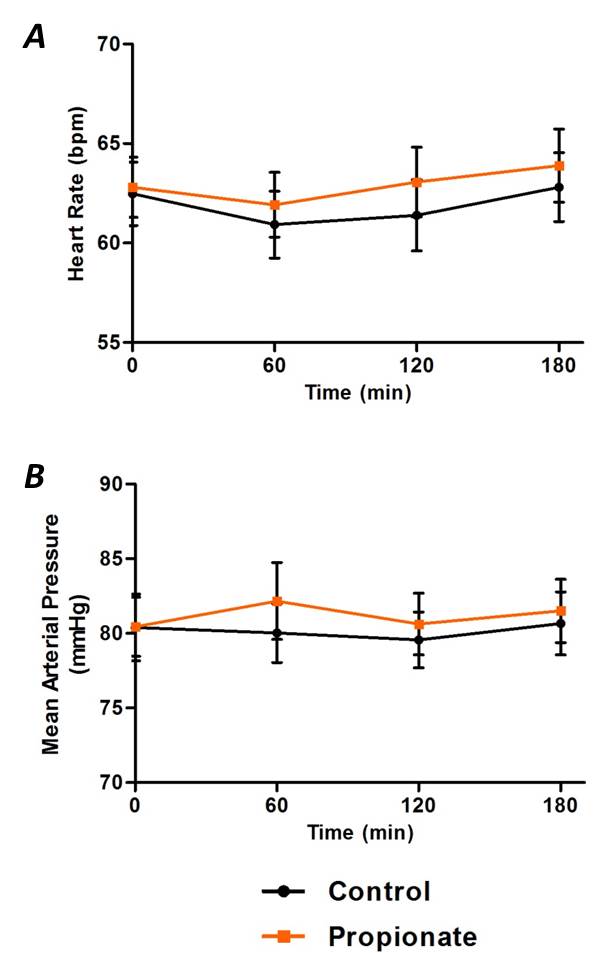
**

**Figure S5. The effect of oral sodium propionate supplementation on heart rate and blood pressure. *A.*** Heart rate (Time×Trial: P= 0.632) and ***B.*** Mean arterial blood pressure (Time×Trial: P= 0.796). All data expressed as mean ± SEM (n=18).


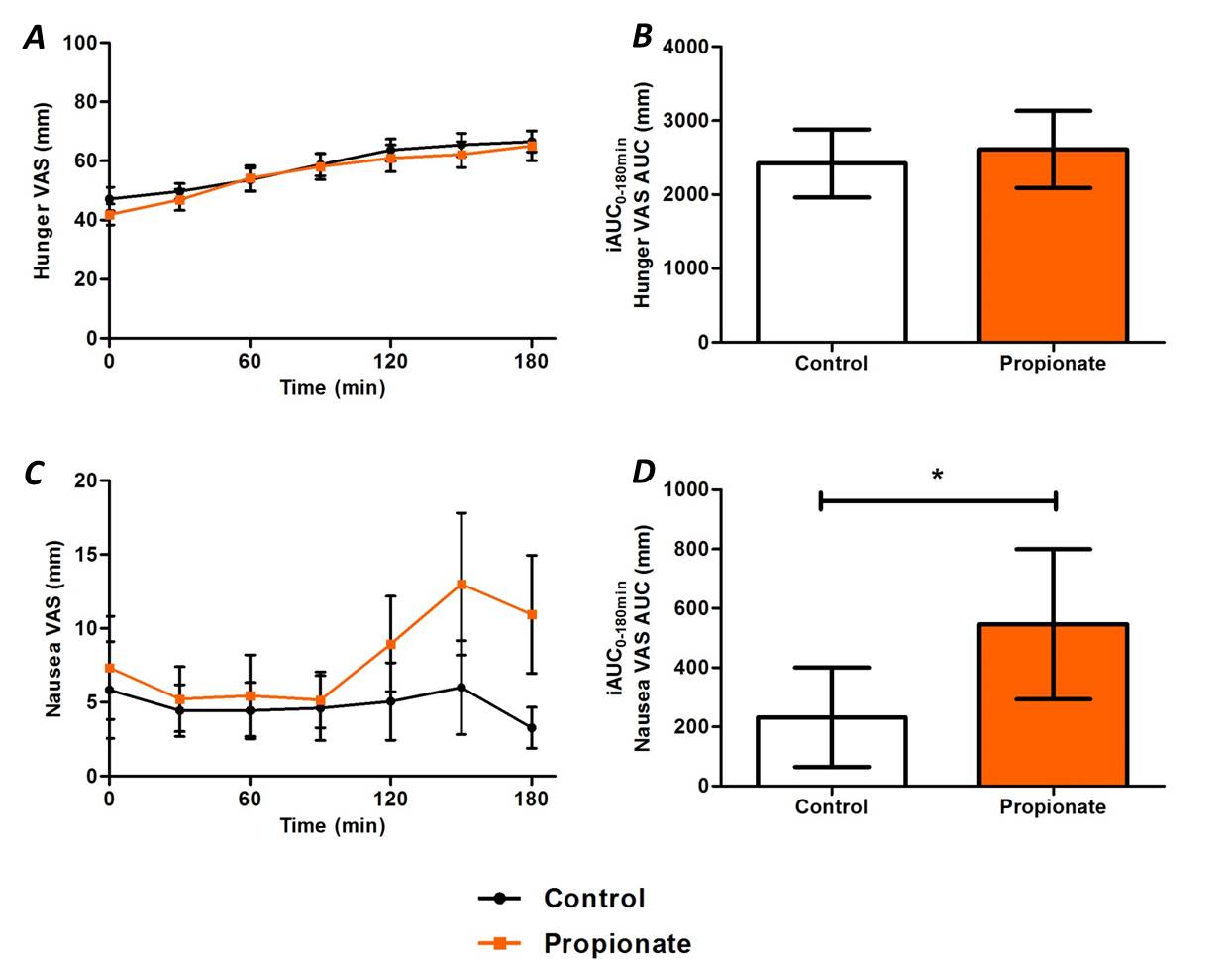


**Figure S6. The effect of oral sodium propionate supplementation on subjective hunger and nausea. *A.*** Hunger (Time×Trial: P= 0.578) and ***B.*** Hunger iAUC (P=0.670). ***C.*** Nausea (Time×Trial: P= 0.073) and ***D.*** Nausea iAUC (P=0.016). All data expressed as mean ± SEM (n=18).

***
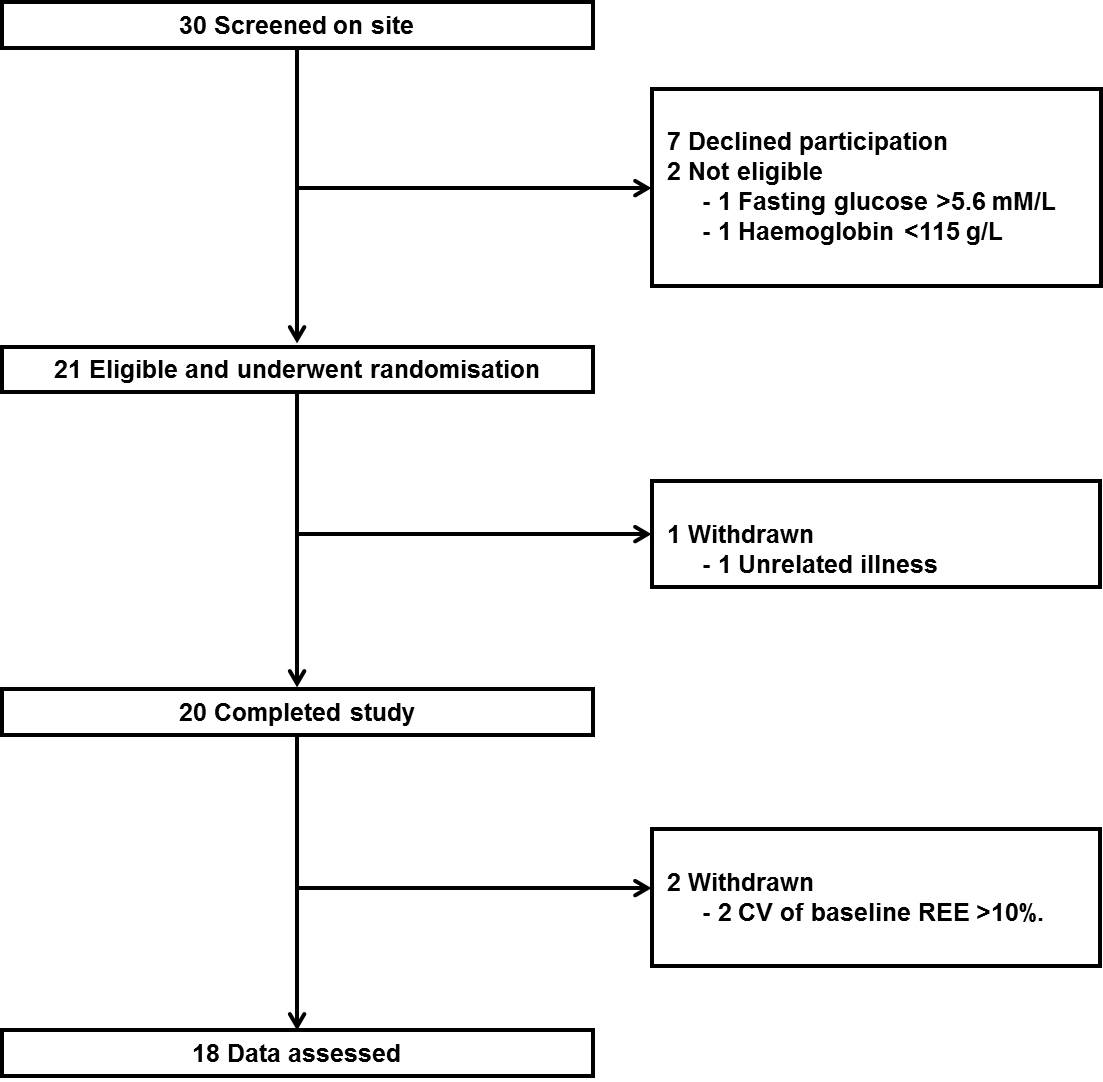
***

**Figure S7. Recruitment and retention in the study.**

***REFERENCES***

**1.** Solomon SJ, Kurzer MS, Calloway DH. Menstrual cycle and basal metabolic rate in women. *Am J Clin Nutr.* Oct 1982;36(4):611-616.

**2.** <http://www.colorcon.com/> CWs. Performance Characteristics of Acryl-EZE®, Aqueous Acrylic Enteric System Accessed August 23, 2017.

**3.** Moreau NM, Goupry SM, Antignac JP, et al. Simultaneous measurement of plasma concentrations and 13C-enrichment of short-chain fatty acids, lactic acid and ketone bodies by gas chromatography coupled to mass spectrometry. *J Chromatogr B Analyt Technol Biomed Life Sci.* Feb 05 2003;784(2):395-403.

**4.** Adrian TE, Ferri GL, Bacarese-Hamilton AJ, Fuessl HS, Polak JM, Bloom SR. Human distribution and release of a putative new gut hormone, peptide YY. *Gastroenterology.* Nov 1985;89(5):1070-1077.

**5.** Donahoo WT, Levine JA, Melanson EL. Variability in energy expenditure and its components. *Curr Opin Clin Nutr Metab Care.* Nov 2004;7(6):599-605.

**6.** Adriaens MP, Schoffelen PF, Westerterp KR. Intra-individual variation of basal metabolic rate and the influence of daily habitual physical activity before testing. *Br J Nutr.* Aug 2003;90(2):419-423.
